# Modulation of AMPK/NLRP3 Signaling Mitigates Radiation-Induced Lung Inflammation by a Synthetic Lipoxin A4 Analogue

**DOI:** 10.3390/ijms262210832

**Published:** 2025-11-07

**Authors:** Sun Ho Min, Jae-Ho Shin, Sunjoo Park, Ronglan Cui, Youn Ji Hur, Woo Hyun Jeong, Sang Yeon Kim, Younghwa Na, Jaeho Cho

**Affiliations:** 1 Department of Radiation Oncology, Yonsei University College of Medicine, Seoul 03722, Republic of Korea; 2 College of Pharmacy, CHA University, Pocheon-si 11160, Gyeonggi-do, Republic of Korea; 3 Department of Food and Nutrition, College of Health Science, Honam University, Gwangju 62399, Republic of Korea

**Keywords:** synthetic lipoxin A4 analogue, CYNC-2, radiation-induced lung inflammation, NLRP3 inflammasome, AMPK pathway

## Abstract

Radiation-induced lung inflammation (RILI) is a major complication of thoracic radiotherapy, characterized by excessive inflammation and subsequent fibrosis that compromise pulmonary function and treatment outcomes. This study explores the pharmacological properties of a newly synthesized Lipoxin A4 analogue (CYNC-2) to mitigate RILI by modulating the AMP-activated protein kinase (AMPK)/NOD-like receptor family pyrin domain containing 3(NLRP3) inflammasome pathway. A murine RILI model was established in mice by delivering a single high-dose (ablative) X-ray irradiation to the left lung. Mice in the treatment group received CYNC-2 via tail-vein injection three times per week for 2 weeks. The effects of CYNC-2 on RILI were evaluated histological, immunohistochemical analysis of lung tissues, cytokine profiling, lung function testing using a FlexiVent system, and micro-computed tomography (micro-CT) imaging of lung damage. In parallel, two human lung cell lines—L132 (normal bronchial epithelial cells) and A549 (lung carcinoma cells)—were irradiated with 6 Gy X-rays and treated with CYNC-2 to assess cell viability and changes in AMPK/NLRP3 pathway markers via qPCR and immunofluorescence. Lung tissue sample from patients who underwent thoracic radiotherapy were also examined to validate key findings. CYNC-2 activated AMPK and inhibited mTOR signaling, which suppressed NLRP3 inflammasome activation and led to reduced secretion of pro-inflammatory cytokines (IL-1β, IL-6, and TGF-β1). In vitro, CYNC-2 mitigated radiation-induced inflammatory responses and preserved cellular viability. Overall, CYNC-2 effectively dampened acute pulmonary in the RILI model. These findings suggest that targeting the AMPK/NLRP3 inflammasome pathway via a stable LXA4 analogue such as CYNC-2 is a promising therapeutic strategy to improve clinical outcomes for patients receiving thoracic radiation therapy.

## 1. Introduction

Thoracic radiotherapy is a cornerstone in the management of lung cancer, breast cancer, and mediastinal malignancies. However, radiation-induced lung injury (RILI), particularly in the form of pneumonitis and fibrosis, remains a significant dose-limiting toxicity that can severely impact patient survival and quality of life [1,2]. RILI is characterized by excessive inflammatory cell infiltration, cytokine release, epithelial and endothelial injury, and subsequent fibrotic remodeling of the lung parenchyma [3].

Among the inflammatory mediators implicated in RILI, the NOD-like receptor protein 3 (NLRP3) inflammasome plays a pivotal role in amplifying radiation-induced inflammation by promoting caspase-1 activation and subsequent maturation of pro-inflammatory cytokines such as interleukin (IL)-1β and IL-18 [4,5]. Aberrant activation of NLRP3 has been associated with progression of pulmonary inflammation and fibrotic remodeling in response to ionizing radiation [6]. Hence, NLRP3 represents a critical therapeutic target for mitigating radiation-induced pulmonary toxicity.

AMP-activated protein kinase (AMPK), a cellular energy sensor, has emerged as a key upstream regulator that negatively controls NLRP3 activation. AMPK suppresses the NLRP3 inflammasome via inhibition of the mTOR/NEK7 axis and induction of autophagy, thereby exerting anti-inflammatory and anti-fibrotic effects [7,8]. However, radiation has been shown to inhibit AMPK signaling while promoting PI3K/Akt/mTOR activity, thus fostering a pro-inflammatory environment in irradiated lung tissue [9]. Therefore, pharmacologic activation of AMPK may counteract radiation-induced dysregulation of immune responses.

Lipoxin A4 (LXA4) is an endogenously derived lipid mediator that plays a central role in the resolution phase of inflammation. It inhibits nuclear factor-κB (NF-κB) and NLRP3 inflammasome activation, promotes macrophage phagocytosis, and accelerates tissue repair [10,11,12,13,14]. Despite its therapeutic potential, native LXA4 has limited clinical utility due to its rapid metabolic degradation, including β-oxidation and 15-hydroxyl oxidation to ketone by 15-hydroxyprostaglandin dehydrogenase [15,16].

To overcome these limitations and prolong its anti-inflammatory efficacy, we designed CYNC-2 as a metabolically stable synthetic analogue of nLXA4. Structurally, CYNC-2 possesses a wider molecular scaffold due to the incorporation of a benzene ring between the diol-bearing chain and a saturated hexyl alcohol moiety, connected via a cis-triene linker. This configuration is expected to confer resistance to enzymatic oxidation. Additionally, we introduced an ether oxygen between the benzene ring and alkyl chain to further enhance metabolic stability.

In this study, we report the synthesis and biological evaluation of CYNC-2 in the context of radiation-induced lung inflammation. Using a clinically relevant murine model of RILI and irradiated human lung epithelial and carcinoma cell lines, we investigated the therapeutic effects of CYNC-2 on inflammatory and fibrotic responses. Our findings demonstrate that CYNC-2 mitigates radiation-induced pulmonary injury by modulating the AMPK/NLRP3 signaling pathway, supporting its further investigation as a candidate compound for RILI management.

## 2. Results

### 2.1. Structural Design and Radioprotective Safety Profile of CYNC-2 in Lung Epithelial Cells

**Figure 1:**
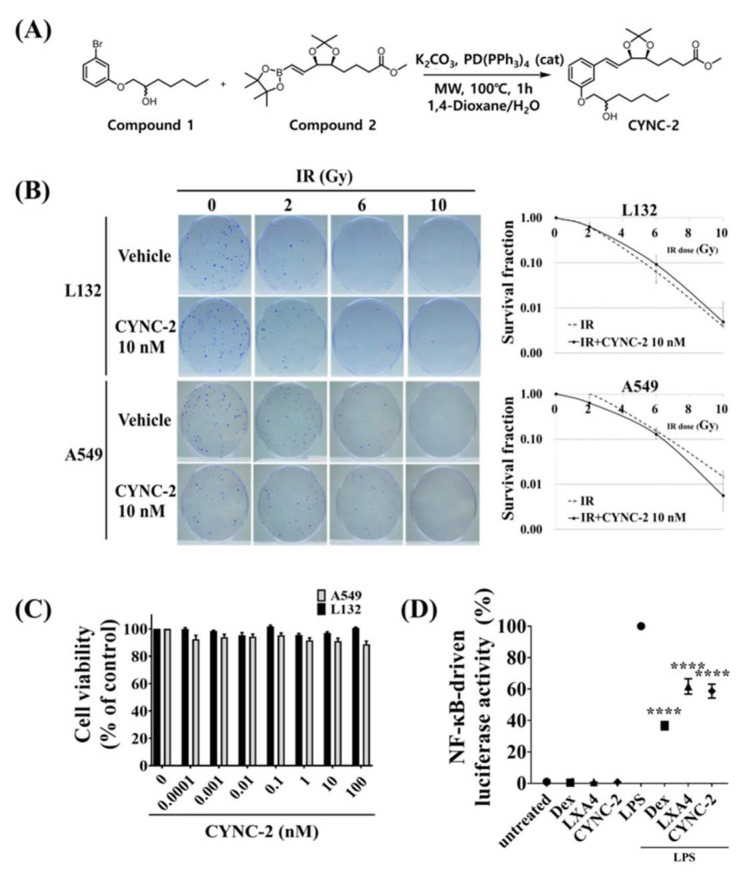
Structural characterization and in vitro evaluation of the radioprotective properties of CYNC-2. (**A**) Chemical structure of the synthetic lipoxin A_4_ analogue CYNC-2. Structural modifications include incorporation of a dioxolane ring system and substitutions at both upper and lower branches to enhance oxidative stability. (**B**) Clonogenic assay evaluating the effects of CYNC-2 (10 nM) on radiation-induced cytotoxicity in normal (L132) and cancerous (A549) lung epithelial cells at doses of 0, 2, 4, 6, and 10 Gy. (**C**) WST-1 cell viability assay performed across a concentration range of CYNC-2 (0.0001–100 nM) in L132 and A549 cells to assess potential cytotoxicity or proliferative effects. (**D**) NF-κB luciferase reporter assay in LPS-stimulated THP-1 Lucia™ monocytes (InvivoGen). Cells were pretreated with CYNC-2, dexamethasone, native LXA_4_, or vehicle control for 2 h prior to stimulation with lipopolysaccharide (LPS, 50 ng/mL). After 24 h, luciferase activity was measured in the culture supernatant using a Lucia detection reagent to quantify NF-κB activation. Dexamethasone was included as a reference compound due to its well-established role as a potent NF-κB inhibitor in in vitro inflammatory assays. Data are presented as mean ± standard deviation. **** *p* < 0.0001.

To address the metabolic instability of native lipoxin A_4_ (nLXA_4_), CYNC-2 was rationally designed as a synthetic analogue with enhanced structural rigidity and oxidative resistance. Structural modifications included substitution of the native open-chain motif with a rigid dioxolane ring system, and modifications to the upper and lower branches of the molecule (Figure 1A). These chemical changes were intended to reduce enzymatic degradation and extend the biological half-life of the compound, as supported by stability analyses (Supplementary Table S1).

To evaluate the cytocompatibility of CYNC-2, WST-1 and clonogenic (CFA) assays were performed in human lung epithelial cell lines: L132 (normal) and A549 (adenocarcinoma). Both cell types were irradiated with increasing doses (0, 2, 4, 6, and 10 Gy) with or without CYNC-2 (10 nM). The survival fraction curves showed no significant differences between irradiated and irradiated + CYNC-2 groups, indicating that CYNC-2 does not exacerbate radiation-induced cytotoxicity (Figure 1B). As expected, survival fractions decreased to ~0.1 at 6 Gy and <0.01 at 10 Gy in both cell types. Interestingly, A549 cells treated with CYNC-2 showed lower viability at 10 Gy than their untreated controls, suggesting a potential tumor-suppressive effect under high-dose radiation conditions.

In addition, to assess the oxidative-stress response, intracellular reactive oxygen species (ROS) levels were measured using a DCFDA-based fluorescence oxidative-stress assay in normal L132 cells. CYNC-2 pretreatment significantly attenuated radiation-induced ROS accumulation, confirming that CYNC-2 mitigates oxidative stress and supports its radioprotective potential in normal lung epithelial cells (Supplementary Figure S1).

A dose–response analysis of CYNC-2 in both L132 and A549 cells revealed minimal changes in viability across a broad concentration range (0.0001–100 nM), indicating low cytotoxicity and no evidence of abnormal proliferative effects in normal lung epithelial cells (Figure 1C).

To assess anti-inflammatory potential, we employed a lipopolysaccharide (LPS)-stimulated NF-κB luciferase reporter assay. CYNC-2 treatment significantly suppressed LPS-induced NF-κB transcriptional activity, comparable to dexamethasone and native LXA_4_ (Figure 1D). This confirms CYNC-2’s ability to attenuate inflammatory signaling via inhibition of NF-κB activation, a key transcription factor involved in RILI pathogenesis.

### 2.2. CYNC-2 Administration Mitigates RILI in Mice

**Figure 2:**
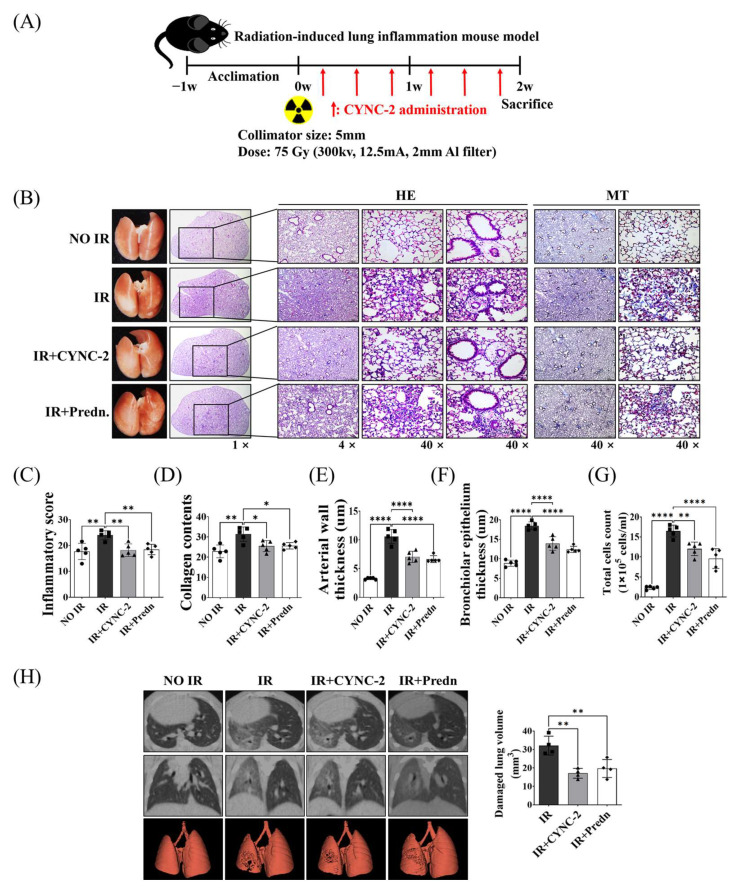
CYNC-2, a novel synthetic LXA4 analogue, attenuates radiation-induced lung injury in vivo. (**A**) Schematic of the experimental timeline for thoracic irradiation and CYNC-2 treatment in mice. (**B**) Representative gross images and lung sections stained with hematoxylin and eosin (H&E) and Masson’s trichrome (MT) at low (1×), intermediate (4×), and high (40×) magnifications. Scale bar = 50 μm. (**C**–**G**) Quantitative histological and cytological analyses: (**C**) Inflammatory score based on infiltrating nuclear cell counts in irradiated lung regions; (**D**) collagen deposition area; (**E**) bronchiolar epithelial thickness; (**F**) arterial wall thickness; (**G**) total inflammatory cell count in bronchoalveolar lavage fluid (BALF), all measured at 2 weeks post-irradiation. (**H**) Micro-computed tomography (micro-CT) images: horizontal (**top**), trans-axial (**middle**), and 3D-reconstructed (**bottom**) views. Predn: Prednisolone (positive control). Data are presented as mean ± standard deviation. * *p* < 0.05, ** *p* < 0.01, **** *p* < 0.0001.

To evaluate the therapeutic efficacy of CYNC-2 in mitigating RILI, lung tissues were collected for histological and molecular analyses two weeks after thoracic irradiation (Figure 2A). Hematoxylin and eosin (H&E) staining of lung sections from irradiated (IR) mice revealed hallmark features of acute radiation injury, including alveolar wall thickening, perivascular and peribronchial inflammatory cell infiltration, and capillary congestion. Masson’s trichrome (MT) staining further demonstrated increased collagen deposition, indicative of early fibrotic remodeling secondary to persistent inflammation. In addition, an in vivo dose–response study was performed using three different CYNC-2 doses (0.5, 2.5, and 5 mg/kg) to confirm the reproducibility and dose robustness of the therapeutic effect. All tested doses significantly reduced radiation-induced inflammatory and fibrotic changes compared with the irradiated control group, with no statistically significant differences among the three doses, indicating a plateau in efficacy (Supplementary Figure S2).

Histological comparison across experimental groups revealed that both CYNC-2- and prednisolone-treated irradiated mice (IR + CYNC-2 and IR + Pred groups, respectively) exhibited marked reductions in alveolar wall thickening, inflammatory infiltration, and vascular congestion relative to untreated irradiated controls (IR group), as shown by H&E staining. MT-stained sections corroborated these findings, showing substantially decreased collagen accumulation in the treatment groups, suggesting attenuation of early fibrogenesis (Figure 2B). Notably, the degree of histological improvement was comparable between the CYNC-2 and prednisolone groups, supporting equivalent anti-inflammatory and anti-fibrotic efficacy.

Quantitative analysis of bronchoalveolar lavage fluid (BALF) parameters provided further validation. The IR group showed significantly increased bronchiolar epithelial thickness, arterial wall thickness, and total inflammatory cell counts compared to the NO IR group. These parameters were significantly normalized in both the IR + CYNC-2 and IR + Pred groups, reaching values not statistically different from those of NO IR group (Figure 2C–G). These results demonstrate that CYNC-2 effectively mitigates radiation-induced pulmonary damage and is comparable in therapeutic potency to prednisolone.

### 2.3. CYNC-2 Preserves Parenchymal Architecture and Improves Lung Function Following Thoracic Irradiation

Micro-computed tomography (micro-CT) imaging revealed structural alterations consistent with radiation-induced lung injury in the IR group, including alveolar collapse, interstitial thickening, and focal fibrotic lesions. In contrast, lungs from CYNC-2- and prednisolone-treated mice exhibited preserved alveolar architecture with minimal fibrotic changes, as shown in horizontal, trans-axial, and three-dimensional reconstructed CT images (Figure 2H). These results confirm that CYNC-2 effectively mitigates radiation-induced functional and structural deterioration of lung tissue.

**Figure 3:**
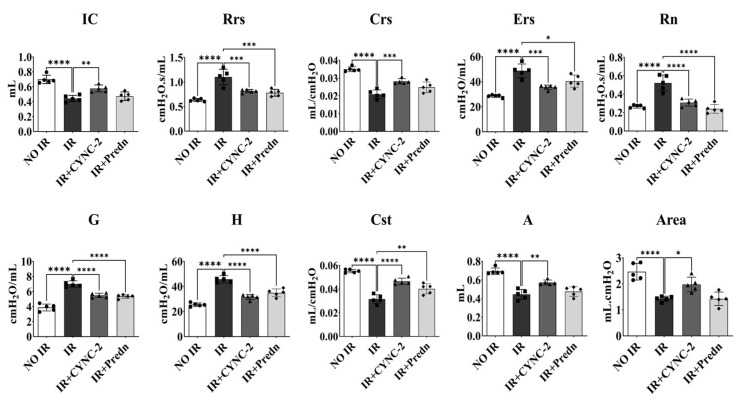
Changes in lung function following CYNC-2 administration. Pulmonary function parameters were assessed using the FlexiVent system, including inspiratory capacity (IC), respiratory system resistance (Rrs), compliance (Crs), elastance (Ers), Newtonian resistance (Rn), tissue damping (G), tissue elastance (H), quasi-static compliance (Cst), tissue damping (A), and hysteresis area of the pressure-volume (P-V) loop (Area). Predn: Prednisolone (positive control). Data are presented as mean ± standard deviation. * *p* < 0.05, ** *p* < 0.01, *** *p* < 0.001, **** *p* < 0.0001.

Furthermore, to assess whether CYNC-2 improves pulmonary mechanics following radiation-induced injury, we performed lung function tests using the FlexiVent system two weeks after irradiation. The irradiated control group (IR) exhibited significantly reduced inspiratory capacity (IC), dynamic and quasi-static compliance (Crs and Cst), and increased respiratory system resistance (Rrs), elastance (Ers), Newtonian resistance (Rn), tissue damping (G), and tissue elastance (H), consistent with impaired pulmonary function (Figure 3). The functional pulmonary parameters evaluated here are listed in Supplementary Table S2.

In contrast, both the IR + CYNC-2 and IR + Prednisolone groups demonstrated significant improvement across most parameters. CYNC-2 treatment restored IC and Cst values close to those of non-irradiated controls (NO IR group), and normalized resistance and elastance values, indicating improved airway and parenchymal function. These functional improvements were comparable to those observed in the prednisolone-treated group, supporting the therapeutic efficacy of CYNC-2 in preserving lung compliance and reducing tissue stiffness post-irradiation.

### 2.4. CYNC-2 Suppresses Radiation-Induced Cytokine Expression in Lung Tissue and Cells

**Figure 4:**
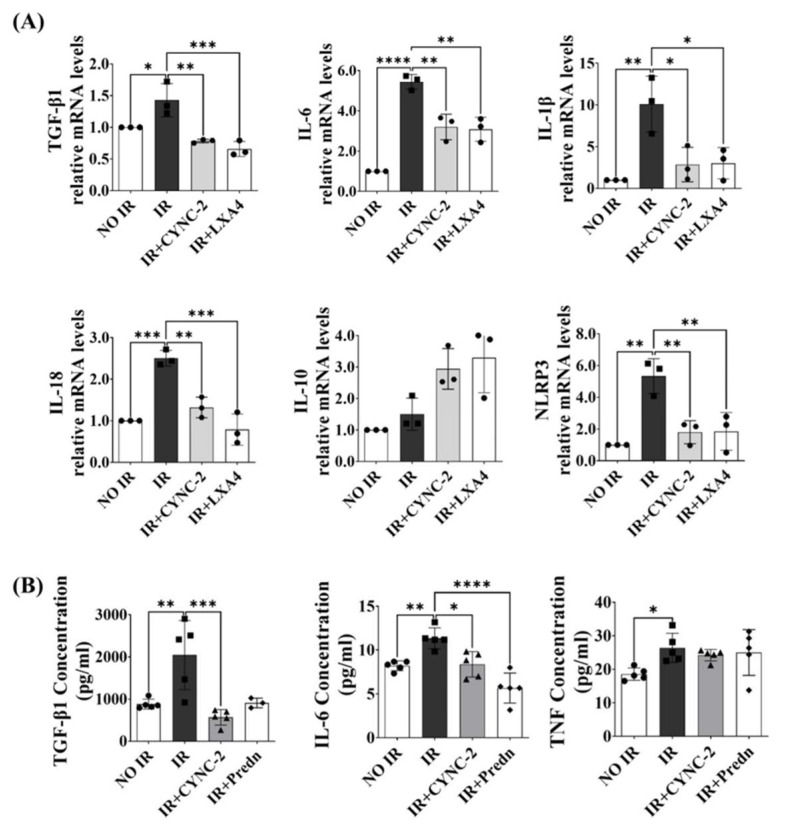
In vitro and in vivo evaluation of the anti-inflammatory effects of CYNC-2. (**A**) Relative mRNA expression of inflammation-related cytokines (IL-1β, IL-6, and TGF-β1) in L132 cells following 6-Gy irradiation with or without CYNC-2 treatment, assessed by quantitative real-time PCR (qRT-PCR). (**B**) Serum levels of the same cytokines measured by ELISA in irradiated mice treated with CYNC-2. Predn: Prednisolone (positive control). Data are presented as the mean ± standard deviation. * *p* < 0.05, ** *p* < 0.01, *** *p* < 0.001, **** *p* < 0.0001.

To evaluate the anti-inflammatory potential of CYNC-2 RILI, cytokine expression levels were measured using quantitative real-time PCR (qRT-PCR) in L132 cells exposed to 6 Gy of ionizing radiation. Treatment with CYNC-2 (10 nM) significantly downregulated the mRNA levels of key pro-inflammatory mediators, including TGF-β1, IL-6, IL-1β, IL-18, and NLRP3. In contrast, the expression of the anti-inflammatory cytokine IL-10 was modestly elevated, although the difference did not reach statistical significance. These effects were comparable to those observed with native LXA4 (nLXA4), supporting the hypothesis that CYNC-2 replicates the endogenous anti-inflammatory activity of LXA4 (Figure 4A).

To further investigate the systemic impact of CYNC-2 in vivo, cytokine levels were quantified in plasma samples from the murine RILI model using enzyme-linked immunosorbent assay (ELISA). Mice in the IR + CYNC-2 group exhibited markedly lower levels of TGF-β1 and IL-6 compared to the IR-only group, while TNF levels remained unaffected. Notably, TGF-β1 levels in CYNC-2-treated irradiated mice were even lower than those in non-irradiated controls, indicating a potent suppressive effect of CYNC-2 on this fibrogenic cytokine (Figure 4B).

Collectively, these results demonstrate that CYNC-2 effectively attenuates radiation-induced inflammation by reducing the expression and systemic release of key pro-inflammatory and profibrotic cytokines.

### 2.5. CYNC-2 Activates AMPK and Inhibits the mTOR/NEK-NLRP3 Inflammasome Pathway

**Figure 5:**
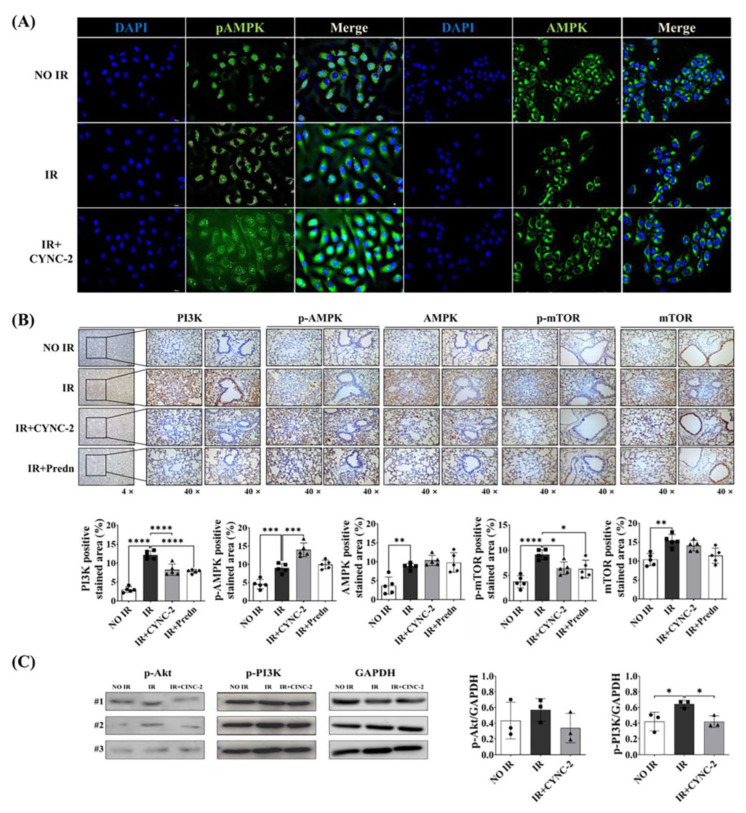
CYNC-2 restores AMPK activation and modulates PI3K/mTOR pathway in irradiated lung tissues and epithelial cells. (**A**) Immunofluorescence staining of L132 cells showing phosphorylated AMPK (pAMPK), total AMPK (green), and nuclei (DAPI, blue) 24 h after 6-Gy irradiation, with or without CYNC-2 pretreatment (1 nM, 2 h prior to IR). CYNC-2 restored pAMPK expression suppressed by irradiation. Images captured at 40× magnification; scale bar = 50 μm. (**B**) Immunohistochemical staining of irradiated mouse lung tissues showing expression of PI3K, pAMPK, total AMPK, p-mTOR, and total mTOR. CYNC-2 treatment increased pAMPK and reduced PI3K and p-mTOR expression. Representative images at 4× and 40× magnification; scale bar = 50 μm. Predn: Prednisolone (positive control). (**C**) Western blotting analysis of P-Akt and p-PI3K expression after in L132 cells exposed to 6 Gy IR. Data are presented as mean ± standard deviation. * *p* < 0.05, ** *p* < 0.01, *** *p* < 0.001, **** *p* < 0.0001.

To elucidate the molecular mechanism underlying CYNC-2’s anti-inflammatory effect, we investigated its impact on the AMPK signaling axis, which plays a critical role in regulating cellular energy homeostasis and inflammation. L132 cells were irradiated with 6 Gy and treated with CYNC-2, followed by immunocytochemical analysis 24 h post-irradiation. Phosphorylated AMPK (pAMPK) expression was significantly reduced in irradiated cells compared to non-irradiated controls, indicating that ionizing radiation suppresses AMPK activation. Notably, CYNC-2 treatment effectively restored pAMPK levels, suggesting that it counteracts radiation-induced inhibition of AMPK activation (Figure 5A).

To validate these findings in vivo, we performed immunohistochemical (IHC) analysis of lung tissues to assess protein expression of pAMPK, total AMPK, PI3K, and phosphorylated mTOR (p-mTOR). CYNC-2 administration markedly increased pAMPK levels in irradiated lung tissues, while PI3K, p-PI3K and p-mTOR expression were significantly reduced (Figure 5B,C). Importantly, total AMPK expression remained unchanged across all groups, indicating that CYNC-2 specifically promotes AMPK phosphorylation rather than altering its overall protein levels.

These results demonstrate that CYNC-2 mitigates radiation-induced lung inflammation by reactivating the AMPK pathway and downregulating PI3K/mTOR signaling. Given the established role of mTOR in activating NEK7 and the NLRP3 inflammasome, these findings suggest that CYNC-2 exerts its therapeutic effects through coordinated suppression of the mTOR–NEK7–NLRP3 axis. Collectively, these data support the therapeutic potential of CYNC-2 in preventing RILI and subsequent fibrotic progression.

### 2.6. CYNC-2 Suppresses NLPR3 Inflammasome Activation in Irradiated Lung Tissue and Cells

**Figure 6:**
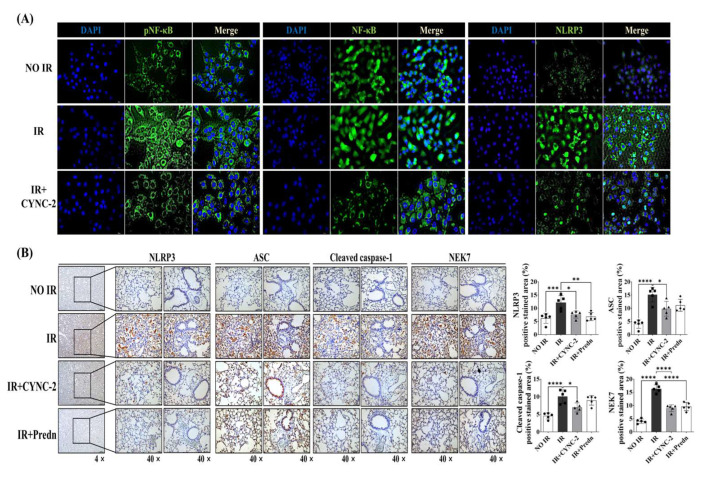
CYNC-2 suppresses radiation-induced NLRP3 inflammasome activation in vitro and in vivo. (**A**) Immunofluorescence analysis of L132 cells showing expression of phosphorylated NF-κB (pNF-κB), total NF-κB, and NLRP3 (green) with nuclear counterstaining (DAPI, blue). Cells were pretreated with CYNC-2 (1 nM) or vehicle for 2 h, followed by 6-Gy X-ray irradiation. Images were captured 24 h post-irradiation at 40× magnification. Scale bar = 50 μm. (**B**) Representative immunohistochemical staining of irradiated mouse lung tissues showing expression of NLRP3, apoptosis-associated speck-like protein containing a CARD (ASC), cleaved caspase-1, and NEK7. CYNC-2 or prednisolone treatment markedly reduced the expression of inflammasome markers compared with the irradiated control group. Images captured at 4× and 40× magnification. Scale bar = 50 μm. Predn: Prednisolone (positive control). Data are presented as mean ± standard deviation. * *p* < 0.05, ** *p* < 0.01, *** *p* < 0.001, **** *p* < 0.0001.

To evaluate the effect of CYNC-2 on NLRP3 inflammasome activation in radiation-induced lung injury (RILI), we first assessed its impact on upstream signaling in irradiated L132 cells. Immunocytochemical analysis performed 24 h post-irradiation revealed that exposure to 6 Gy significantly increased phosphorylated NF-κB (pNF-κB), total NF-κB, and NLRP3 expression levels. CYNC-2 pretreatment markedly attenuated the radiation-induced upregulation of these proteins, suggesting that it mitigates inflammatory signaling at both transcriptional and inflammasome activation levels (Figure 6A).

We next examined the in vivo effect of CYNC-2 on inflammasome components in irradiated lung tissues. Immunohistochemical staining showed that expression of NLRP3, apoptosis-associated speck-like protein containing a CARD (ASC), and cleaved caspase-1 was significantly increased in the IR group, consistent with inflammasome activation. Notably, treatment with CYNC-2 or prednisolone substantially reduced the positively stained areas for these proteins to levels comparable to the non-IR group. Although NEK7 expression was not directly targeted by CYNC-2, its reduced staining in the CYNC-2 and prednisolone groups suggests a possible upstream modulation of the NLRP3 activation cascade (Figure 6B).

Collectively, these findings indicate that CYNC-2 effectively suppresses radiation-induced NLRP3 inflammasome activation, likely through inhibition of upstream NF-κB signaling and downstream ASC–caspase-1 axis. This mechanism contributes to the observed anti-inflammatory and tissue-protective effects of CYNC-2 in the RILI model.

### 2.7. NLRP3 Upregulation in Patients Lung Tissues Highlights Its Clinical Relevance in RILI

**Figure 7:**
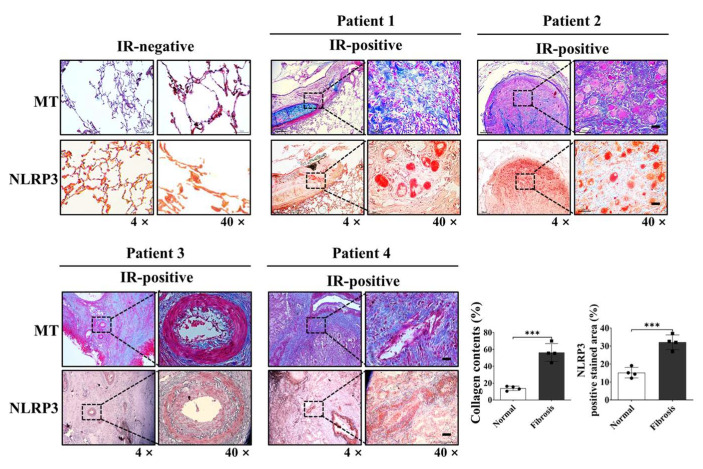
Clinical validation of NLRP3 upregulation in human lung tissue following thoracic radiotherapy. Representative histological sections from n = 4 patients with locally advanced lung cancer who underwent neoadjuvant concurrent chemoradiotherapy (CCRT) followed by surgical resection. Masson’s trichrome staining revealed enhanced collagen accumulation in irradiated lung regions, indicating active fibrotic remodeling, while immunohistochemical staining showed co-localized upregulation of NLRP3 expressions in the same regions. Quantitative analysis of collagen-positive and NLRP3-positive area fractions was performed using ImageJ, and results are shown as bar graphs (mean ± SD). Images were captured at 4× and 40× magnification. Scale bar = 50 μm. See Supplementary Table S3 for detailed clinical and treatment information for each patient. *** *p* < 0.001.

To evaluate the clinical relevance of NLRP3 inflammasome activation in radiation-induced lung injury (RILI), we analyzed lung specimens obtained from four patients with locally advanced lung cancer who had undergone neoadjuvant concurrent chemoradiotherapy (CCRT) followed by surgery. This treatment approach, though relatively uncommon, enabled access to lung tissues from irradiated fields. The total radiation dose (45–54 Gy) and the interval between completion of radiotherapy and surgical resection (4–8 weeks) are summarized in Supplementary Table S3. Masson’s trichrome (MT) and immunohistochemical (IHC) staining were performed to assess collagen deposition and NLRP3 expression, respectively. MT staining revealed marked collagen accumulation in irradiated lung tissues, indicating active fibrotic remodeling. Correspondingly, IHC analysis demonstrated significant upregulation of NLRP3 expression in areas affected by radiation. Importantly, NLRP3-positive regions co-localized with zones of enhanced fibrosis and inflammatory cell infiltration, underscoring the spatial association between NLRP3 activation and pathological lung remodeling. To provide objective support, quantitative analysis of the MT- and NLRP3-positive area fractions was conducted using ImageJ, and the results are presented as bar graphs in Figure 7, alongside representative histological images. A positive correlation trend was observed between radiation dose and the IHC-positive staining area for NLRP3, indicating that higher radiation exposure may be associated with increased NLRP3 expression. 

These findings validate the role of NLRP3 inflammasome activation in human RILI and support its potential as a clinically relevant therapeutic target for mitigating radiation-induced pulmonary inflammation and fibrosis (Figure 7).

### 2.8. Schematic Summary of CYNC-2 Mechanism Underlying the Effect of CYNC-2 on RILI

**Figure 8:**
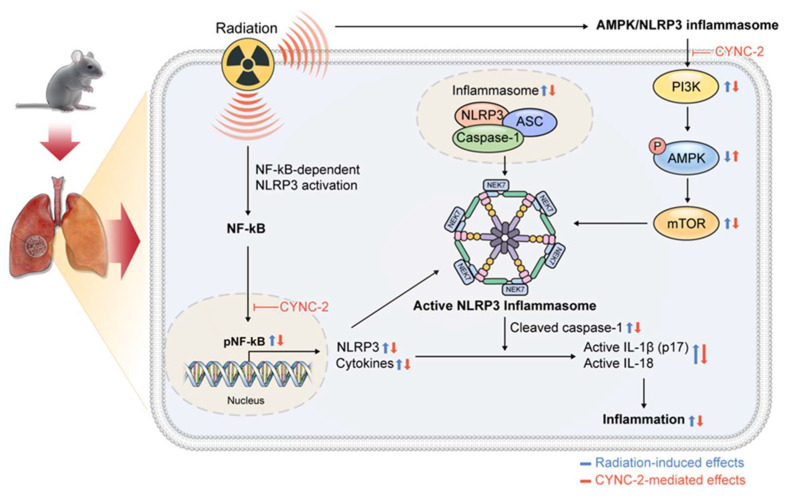
Proposed mechanism of CYNC-2 in attenuating radiation-induced lung injury (RILI). Schematic illustration of the signaling pathway modulated by CYNC-2 in the context of RILI. CYNC-2 inhibits PI3K activity, leading to activation of AMPK and suppression of mTOR signaling. This downregulates NEK7 expression, thereby impairing NLRP3 inflammasome assembly and reducing caspase-1 activation. The downstream effects include decreased maturation and secretion of pro-inflammatory cytokines IL-1β and IL-18, ultimately mitigating lung inflammation remodeling.

The proposed mechanism underlying the therapeutic effect of CYNC-2 involves modulation of the AMPK/NLRP3 inflammasome signaling cascade. CYNC-2 inhibits PI3K activity, leading to enhanced phosphorylation and activation of AMPK. Activated AMPK subsequently suppresses mTOR signaling, which is associated with reduced NEK7 expression. This downregulation of NEK7 impairs NLRP3 inflammasome assembly and activation, resulting in decreased caspase-1 cleavage. Consequently, the maturation and secretion of pro-inflammatory cytokines IL-1β and IL-18 are diminished, ultimately attenuating the inflammatory response characteristic of radiation-induced lung injury. (Figure 8).

## 3. Discussion

This study demonstrates that CYNC-2, a newly developed synthetic lipoxin A4 analogue, effectively attenuates RILI by modulating the AMPK/NLRP3 inflammasome signaling axis. In a murine model of RILI, CYNC-2 treatment significantly reduced inflammatory cell infiltration, collagen deposition, and functional impairment, indicating strong anti-inflammatory activity. It is important to note that the discrepancy between radiation doses used in the in vitro and in vivo experiments reflects fundamental differences in experimental context and biological responsiveness. Previous studies have established that cultured human epithelial cells, including A549 and L132, typically respond to single-fraction ionizing radiation at doses ranging from 2 to 10 Gy, which are sufficient to induce cytotoxicity, DNA damage, and pro-inflammatory signaling in a controlled environment [17,18,19]. In contrast, preclinical animal models particularly those designed to mimic clinical stereotactic body radiotherapy (SBRT) often require substantially higher focal doses (e.g., 70–90 Gy) to elicit localized pulmonary injury and fibrotic remodeling within a tractable experimental window [20,21,22]. This is especially relevant in partial-lung irradiation models like ours, which provide improved clinical relevance by targeting a defined lung volume analogous to human radiotherapy fields, as previously demonstrated [21,22]. Moreover, the higher radio resistance and regenerative capacity of rodent lung tissue necessitate the use of such ablative doses to reproduce the key pathological features of human RILI. Therefore, the use of 6 Gy in cell-based assays and 75 Gy in focal lung irradiation represents a deliberate and validated experimental design tailored to each model’s biological sensitivity and research purpose.

The NLRP3 inflammasome plays a pivotal role in the pathogenesis of RILI by activating caspase-1 and promoting the maturation and secretion of pro-inflammatory cytokines IL-1β and IL-18 [6,23,24,25]. In line with previous studies showing that radiation amplifies pulmonary inflammation via NLRP3 activation [6,26], our data showed that CYNC-2 markedly suppressed NLRP3 expression and downstream cytokine production in both murine tissue and irradiated lung epithelial cells. Furthermore, histological evidence of decreased collagen accumulation suggests that CYNC-2 has the potential to mitigate fibrotic progression. These findings align with existing literature on the therapeutic potential of targeting NLRP3 in radiation-induced injury [23,24,25,26,27].

Importantly, CYNC-2’s efficacy was comparable to that of prednisolone, a standard anti-inflammatory agent, as observed in multiple histopathological and quantitative parameters. Given the well-known adverse effects associated with long-term corticosteroid use, CYNC-2 may offer a targeted and potentially safer alternative therapeutic strategy for RILI management [28]. CYNC-2 also suppressed IL-6 and TGF-β1 levels, cytokines implicated in chronic inflammation, further supporting its immunomodulatory potential [26]. While our study employed prednisolone as a positive control to reflect current clinical management of RILI, future studies using pathway-specific agents such as AICAR and MCC950 are warranted to delineate the precise mechanistic role of the AMPK/NLRP3 axis in CYNC-2’s therapeutic activity. Inclusion of such controls would provide deeper insight into whether CYNC-2’s efficacy is primarily mediated through AMPK activation, inflammasome inhibition, or both.

Mechanistically, CYNC-2 exerts its therapeutic effect by modulating the PI3K–AMPK–mTOR–NEK7–NLRP3 signaling axis. Our findings indicate that CYNC-2 inhibits PI3K activity, leading to AMPK activation and subsequent mTOR suppression. This cascade reduces NEK7 expression, which is necessary for NLRP3 inflammasome assembly, ultimately diminishing caspase-1 activation and the release of IL-1β and IL-18. Although our study did not directly assess NEK7 activity, its downstream influence in this signaling pathway highlights a plausible target for future mechanistic investigation. This proposed cascade underscores CYNC-2’s ability to intersect inflammatory and metabolic regulatory networks, which are crucial in the progression of radiation-induced injury [9,29].

The observed reduction in radiation-induced ROS levels demonstrates that CYNC-2 effectively mitigates oxidative stress in normal lung epithelial cells, further supporting its role as a potential radioprotective and anti-inflammatory agent.

The therapeutic implications of these findings extend to clinical settings. Our analysis of patient-derived lung tissue samples demonstrated co-localization of collagen deposition and NLRP3 expression, reinforcing the clinical relevance of this target. By dampening both acute inflammatory and chronic fibrotic responses, CYNC-2 may preserve lung function and mitigate long-term complications in patients receiving thoracic radiotherapy. Comparatively, other anti-fibrotic agents such as ACT001 and PM014 have shown similar efficacy in preclinical models [30,31], suggesting that CYNC-2 belongs to a promising class of radiation countermeasures.

Beyond RILI, the dual anti-inflammatory and anti-fibrotic properties of CYNC-2 may have potential applications in other chronic pulmonary disorders, including idiopathic pulmonary fibrosis (IPF), where aberrant inflammasome activation and unresolved inflammation are critical pathophysiological features [32]. Moreover, previous studies on native lipoxins support the suppression of TGF-β1 and IL-1β signaling as a conserved anti-inflammatory mechanism [33,34], which CYNC-2 appears to emulate with enhanced stability and potency.

Nevertheless, the current study has limitations. Our findings are based primarily on a preclinical murine model and in vitro analyses. While the results are encouraging, further studies are needed to establish optimal dosing regimens, evaluate long-term efficacy, and validate safety profiles in larger animal models or clinical settings. Extended follow-up using advanced imaging techniques such as micro-CT may also offer better insights into fibrosis progression and therapeutic response. Comprehensive toxicity studies, including determination of LD50, would also be necessary in future investigations during the formal drug development process.

We demonstrated that CYNC-2 alleviates radiation-induced pulmonary inflammation via modulation of the AMPK–NLRP3 signaling pathway, based on data derived from both immune (THP-1) and structural lung cell types (L132), as well as in vivo models. To evaluate this mechanism across different cellular contexts, we initially employed monocyte-derived reporter cells to screen for NF-κB inhibition and subsequently validated these findings in human lung epithelial cells. This sequential approach provided complementary evidence from immune and parenchymal systems, supporting the robustness of CYNC-2’s mechanism of action. While these models represent distinct cellular environments, the convergent inhibition of NF-κB activation observed in both suggests that CYNC-2 modulates a shared inflammatory signaling axis. Nevertheless, because these results were obtained in preclinical models using distinct cell types, direct translation to human pathophysiology or therapeutic application should be approached with caution.

We acknowledge that only four human samples were analyzable and all patients received similar radiation doses (45–54 Gy) as part of neoadjuvant chemoradiotherapy. Because of this narrow and protocol-defined dose range, a statistical correlation analysis between radiation dose and NLRP3 expression was not feasible. Nevertheless, the consistent NLRP3 upregulation observed in fibrotic areas across these cases supports its biological relevance. Supplementary Table S3 provides full clinical context. In addition, since CYNC-2 was administered systemically, there is potential for unintended effects on healthy tissue cells and the immune system. While dose-finding studies are typically conducted prior to efficacy testing, the initial in vivo dose (0.5 mg/kg) in this study was selected based on prior optimization with a structurally related analogue (CYNC-1), which had demonstrated maximal anti-inflammatory efficacy without toxicity in a comparable model. In accordance with the 3Rs principle, the primary efficacy experiments were therefore conducted at this biologically plausible dose, followed by a complementary dose-ranging analysis (0.5–5 mg/kg) to confirm the robustness and plateau of therapeutic efficacy (Supplementary Figure S2). Future studies should therefore investigate the safety profile of CYNC-2, including its long-term effects, tissue-specific responses, and intercellular interactions using co-culture and in vivo models to confirm translational applicability.

In conclusion, this study provides compelling preclinical evidence that CYNC-2 is a promising therapeutic agent for RILI. By modulating the AMPK/NLRP3 signaling pathway, CYNC-2 alleviates inflammation, preserves pulmonary function, and offers a mechanistically distinct and potentially safer alternative to corticosteroids. Future research should focus on translating these findings to clinical applications for patients undergoing thoracic radiation therapy.

## 4. Materials and Methods

### 4.1. Animal Experiment

Male C57BL/6 mice (6 weeks old) were purchased from Central Lab Animal Inc. (Seoul, Republic of Korea). All experimental protocols were approved by the Institutional Animal Care and Use Committee of Yonsei University Health System (YUHS-IACUC; approval no. 2022-0186) and performed in accordance with relevant ethical guidelines and regulations. Radiation was delivered using the X-RAD 320 platform (Precision X-ray, North Branford, CT, USA) following previously established protocols [35]. Mice were randomly assigned to one of the following four groups (n = 5 per group): (1) non-irradiated control group, (2) irradiated (IR) group, (3) IR + CYNC-2 treatment group, and (4) IR + prednisolone treatment group. To induce RILI, mice received a single 75 Gy dose of localized irradiation to the left lung using a 5 mm collimator. CYNC-2 (0.5 mg/kg) or prednisolone (2 mg/kg) was administered via tail vein injection three times per week for two weeks following irradiation. Lung tissues were harvested two weeks after IR for histological, immunological, and functional analyses.

The initial in vivo dose of CYNC-2 (0.5 mg/kg) was guided by prior efficacy and tolerability data from a structurally related analogue (CYNC-1), which had demonstrated maximal anti-inflammatory activity without toxicity in a comparable model. In accordance with the 3Rs principle, the primary efficacy study was conducted using this biologically plausible dose, and a complementary dose-ranging experiment was subsequently performed to verify dose robustness.

### 4.2. Synthesis of the Novel Synthetic Lipoxin A4 Analogue (CYNC-2)

CYNC-2 was synthesized via Suzuki coupling using 3-bromophenoxyalcohol (compound 1) and a boronic acid ester (compound 2). Compound 1 was obtained through a two-step reaction starting from 3-bromophenol, and compound 2 was synthesized based on a modified protocol [36]. The reaction involved 100 mg of compound 1 (0.35 mmol), 185 mg of compound 2 (0.52 mmol), and 9.6 mg of K_2_CO_3_ (0.07 mmol) in 5 mL 1,4-dioxane and 1 mL water. The mixture was heated at 100 °C in a microwave reactor with tetrakis(triphenylphosphine)palladium(0) (5 mg, 5 wt%) under argon. After extraction and purification, CYNC-2 was obtained as a transparent liquid (yield: 36.9%). Its structure was confirmed by HPLC (purity: 98.5%), 1H-NMR, 13C-NMR, and HRMS (*m*/*z* [M + Na]+ calcd 457.2563, found 457.2561).

### 4.3. Histological and Immunohistochemical (IHC) Analysis

Mouse lung tissues were sectioned at a thickness of 4 μm and subjected to histological staining with hematoxylin and eosin (H&E) and Masson’s trichrome (MT) to assess inflammation and fibrotic remodeling. For immunohistochemistry, the following primary antibodies were used: anti-PI3K (1:300, #ab189403, Abcam, Cambridge, UK), anti-phospho-AMPK (p-AMPK, 1:300, #2532, Cell Signaling Technology, Danvers, MA, USA), anti-AMPK (1:300, #ab32047, Abcam), anti-phospho-mTOR (p-mTOR, 1:500, #GTX132803, GeneTex, Zeeland, MI, USA), anti-mTOR (1:500, #GTX101557, GeneTex), anti-NLRP3 (1:300, #GTX00763, GeneTex), anti-TMS1/ASC (1:500, #GTX55818, GeneTex), anti-cleaved caspase-1 (1:200, #2532, Cell Signaling Technology), and anti-NEK7 (1:400, #GTX636769, GeneTex). Tissue sections were incubated with primary antibodies followed by appropriate HRP-conjugated secondary antibodies, and colorimetric detection was performed using 3,3’-diaminobenzidine (DAB). Quantification of positively stained areas was conducted using ImageJ software (version 1.51; NIH, Bethesda, MD, USA) with the color deconvolution plugin. The software was obtained from https://imagej.net/ij/download.html (accessed on 30 October 2023).

### 4.4. NF-κB Reporter Gene Assay

To evaluate NF-κB activation, we employed the THP1-Lucia™ NF-κB monocyte reporter cell line (cat# thpl-nfkbv2, InvivoGen, San Diego, CA, USA), which is stably engineered to secrete Luica luciferase under the control of an NF-κB-responsive promoter. This system enables real-time, non-destructive quantification of NF-κB activity in response to pro-inflammatory stimuli and is widely used in innate immunity research due to the high responsiveness of monocytes to TLR ligands. Cells were maintained in RPMI-1640 medium supplemented with 2 mM L-glutamine, 25 mM HEPES, 10% fetal bovine serum (FBS; Welgene, Gyeongsan, Republic of Korea), 100 U/mL penicillin, 100 μg/mL streptomycin, 100 μg/mL Normocin, and 100 μg/mL Zeocin, following manufacturers’ instructions. For the assay, cells were seeded at a density of 1 × 10^5^ cells per well in 96-well plates containing culture medium with 0.1% FBS. Following a 2 h pre-treatment with lipoxin A_4_ (LXA_4_; 1 nM), dexamethasone (1 μM), or vehicle control (0.1% DMSO), cells were stimulated with lipopolysaccharide (LPS; 100 ng/mL) for 24 h. Cell culture supernatants were collected, and luciferase activity was measured using a coelenterazine-based luminescence assay (Quanti-Luc™; InvivoGen) on a microplate reader (VersaMax, Molecular Devices, San Jose, CA, USA). Luminescence intensity was used as a direct readout of NF-κB transcriptional activity.

### 4.5. Micro-Computed Tomography (Micro-CT)

Micro-computed tomography (micro-CT) imaging was performed to assess structural changes in irradiated lung tissue. High-resolution scans were acquired using the Quantum GX2 micro-CT system (PerkinElmer, Shelton, CT, USA). Scanning parameters were set as follows: 50 kVp tube voltage, 180 μA tube current, and 150 mGy radiation dose. Two-dimensional (2D) axial images (resolution: 1132 × 1120 pixels) were obtained and reconstructed into three-dimensional (3D) datasets. The extent of radiation-induced pulmonary fibrosis was quantitatively assessed using Analyze 14.0 software (AnalyzeDirect, Overland Park, KS, USA), which provides automated segmentation and computation of structural indices based on both 2D and 3D CT images.

### 4.6. FlexiVent™ Pulmonary Function Analysis

Pulmonary mechanics were evaluated in irradiated mice using the FlexiVent™ system (SCIREQ, Montreal, QC, Canada), a computer-controlled ventilator specifically designed for small animals. This system enables precise measurement of respiratory parameters, including airway resistance, lung compliance, and forced oscillation mechanics. Mice were anesthetized and tracheostomized prior to connection to the ventilator. Quasi-sinusoidal ventilation was applied with a tidal volume of 10 mL/kg at a respiratory rate of 150 breaths/min. Measurements were initiated following the stabilization of ventilation to minimize spontaneous breathing artifacts. Each perturbation maneuver was repeated sequentially, and three technically valid measurements (coefficient of determination > 0.95) per mouse were obtained. The average values were used for downstream analyses.

### 4.7. Bronchoalveolar Lavage Fluid (BALF) Analysis

After euthanasia, bronchoalveolar lavage was performed by inserting a 1 mL syringe filled with phosphate-buffered saline (PBS) into the exposed trachea. A total of 1 mL PBS was instilled into the lungs and gently aspirated back. This procedure was repeated three times to maximize fluid recovery. The recovered BALF was centrifuged at 1300 rpm for 10 min at 4 °C. Cell pellets were resuspended in 1 mL PBS and applied to cell counting slides. Total cell counts were performed using an automated cell counter (EVE™, NanoEntek, Seongnam-si, Gyeonggi-do, Republic of Korea).

### 4.8. Cell Culture and Treatment

Two human lung-derived cell lines were employed in this study: L132 (normal lung epithelial cells; ATCC, Manassas, VA, USA) and A549 (lung adenocarcinoma cells; Korean Cell Line Bank, Seoul, Republic of Korea). Cells were maintained in Dulbecco’s Modified Eagle Medium (DMEM; Welgene, Gyeongsan-si, Republic of Korea) supplemented with 10% fetal bovine serum (FBS) and incubated at 37 °C in a humidified atmosphere containing 5% CO_2_. Cells were seeded at a density of 0.5 × 10^6^ cells per 100 mm culture dish and incubated for 24 h to allow adherence. Prior to experimental treatments, cells were serum-starved overnight under serum-free DMEM conditions.

### 4.9. Cell Irradiation

Cells were irradiated using the X-RAD 320 X-ray irradiator (Precision X-Ray, North Branford, CT, USA) at a dose rate of 1.6 Gy/min. A total dose of 6 Gy was administered, based on previously established protocols [37].

### 4.10. WST-1 Cell Survival Assay

The viability of L132 and A549 cells was assessed using the WST-1 Assay Kit (Dojindo, Kagoshima, Japan) in accordance with the manufacturer’s protocol. Cells were seeded at a density of 1 × 10^4^ cells per well in 96-well plates containing 100 µL of complete medium and were allowed to adhere overnight at 37 °C in a humidified atmosphere with 5% CO_2_. After treatment, 10 µL of WST-1 reagent was added to each well, and the plates were incubated for 2 h. Absorbance was measured at 450 nm (with 620 nm as the reference wavelength) using a microplate reader (VersaMax, Molecular Devices, San Jose, CA, USA). Cell viability was expressed as a percentage of vehicle-treated control cells. All assays were performed in triplicate and repeated independently three times to ensure reproducibility.

### 4.11. Colony Formation Assay

Clonogenic potential was evaluated in irradiated L132 and A549 cells using a colony formation assay (CFA). Cells were seeded in 60 mm culture dishes and exposed to ionizing radiation at doses of 2, 6, or 10 Gy using the X-RAD 320 system (Precision X-Ray). Following irradiation, cells were cultured in DMEM supplemented with 10% FBS for 14 days under standard conditions (37 °C, 5% CO_2_).

After the incubation period, colonies were fixed with cold methanol for 30 min and stained with 0.5% crystal violet. Colonies consisting of ≥50 cells were counted manually, and the surviving fraction was calculated by normalizing to the plating efficiency of non-irradiated control cells.

### 4.12. Quantitative Real-Time PCR (qRT-PCR)

Total RNA was extracted from cells using TRIzol reagent (Thermo Fisher Scientific, Waltham, MA, USA), and RNA concentration was quantified using a NanoDrop 2000 spectrophotometer (Thermo Fisher Scientific, Waltham, MA, USA). First-strand cDNA synthesis was performed with 1 µg of total RNA using the High-Capacity cDNA Reverse Transcription Kit (Applied Biosystems, Foster City, CA, USA), following the manufacturer’s protocol. Quantitative real-time PCR was conducted using the iQ SYBR Green Supermix (Bio-Rad, Hercules, CA, USA) in a total reaction volume of 20 µL. Primer sequences are provided in Supplementary Table S4. Gene expression was normalized to GAPDH, and relative expression levels were calculated using the ΔΔCT method.

### 4.13. Immunofluorescence Staining

Cells were fixed with 4% paraformaldehyde for 15 min at room temperature, followed by permeabilization with 0.1% Triton X-100 in phosphate-buffered saline (PBS) for 10 min. After PBS washes, cells were blocked with 5% bovine serum albumin (BSA) in PBS for 1 h at room temperature. Primary antibodies were diluted in 1% BSA/PBS and incubated overnight at 4 °C. The antibodies used were anti-pAMPK (1:200, #ab23875, Abcam), anti-AMPK (1:200, #ab23047, Abcam), anti-pNF-κB (1:200, #3033, Cell Signaling Technology), anti-NF-κB (1:200, #8242, Cell Signaling Technology), and anti-NLRP3 (1:200, #GTC00763, GeneTex). After washing, cells were incubated with Alexa Fluor 488-conjugated secondary antibody (1:1000, #A21206, Invitrogen, Waltham, MA, USA) for 1 h at room temperature in the dark. Nuclei were counterstained with DAPI (1:1000, #8961S, Cell Signaling Technology). Slides were mounted using fluorescence mounting medium (Dako, Troy, MI, USA) and imaged using a BX53 fluorescence microscope (Olympus, Melville, NY, USA).

### 4.14. Enzyme-Linked Immunosorbent Assay (ELISA)

Levels of TGF-β1 (#DY1679, R&D Systems, Minneapolis, MN, USA), IL-6 (#555240, BD Biosciences, Franklin Lakes, NJ, USA), and TNF-α (#560478, BD Biosciences) were quantified using ELISA kits according to the manufacturers’ instructions. Absorbance was measured in triplicate using a microplate reader (VersaMax, Molecular Devices, San Jose, CA, USA).

### 4.15. Human Tissue Analysis

Formalin-fixed, paraffin-embedded lung tissue samples from patients with radiation-induced lung injury (RILI) were obtained under Institutional Review Board approval (Yonsei University Health System, IRB No. 4-2014-0193). Immunohistochemistry (IHC) was performed using either 3-amino-9-ethylcarbazole (AEC) or 3,3′-diaminobenzidine (DAB) as chromogens.

### 4.16. Western Blot Analysis

Cells were lysed in RIPA buffer (50 mM Tris-HCl, pH 7.4; 1% Nonidet P-40; 0.25% sodium deoxycholate; 150 mM NaCl; 1 mM Na_3_VO_4_) supplemented with protease inhibitors (2 mM phenylmethylsulfonyl fluoride, 100 μg/mL leupeptin, 10 μg/mL pepstatin, 1 μg/mL aprotinin, and 2 mM EDTA) and a phosphatase inhibitor cocktail (GenDEPOT, Baker, TX, USA). After incubation for 30 min, the lysates were centrifuged at 14,000 rpm for 20 min at 4 °C, and the supernatants were collected for Western blot analysis. Protein concentrations were determined using a BCA protein assay kit (Bio-Rad, Hercules, CA, USA). Equal amounts of protein (30–40 µg) were separated by SDS–PAGE, transferred onto polyvinylidene fluoride membranes (GE Healthcare, Little Chalfont, UK), and blocked with 5% skim milk for 1 h at room temperature. The membranes were incubated with anti-phospho-Akt (1:1000, #4060, Cell Signaling Technology) and anti-phospho-PI3K (1:500, #GTX132597, GeneTex) antibodies. Protein bands were visualized using an enhanced chemiluminescence detection kit (Thermo Fisher Scientific, Danvers, MA, USA). Western blotting was performed in three independent experiments, and the band intensities were quantified using ImageJ software (NIH, Bethesda, MD, USA)

### 4.17. Statistical Analysis

Statistical analysis was performed using SPSS version 25.0 (IBM SPSS Inc., Chicago, IL, USA). Student’s *t*-test was used for comparisons between two groups, and one-way analysis of variance (ANOVA) followed by Tukey’s post hoc test was used for comparisons among multiple groups. Correlation analysis between radiation dose and NLRP3 IHC staining was performed using Spearman’s rank correlation due to the small sample size (n = 4) and non-normal data distribution. A linear regression line was plotted for visualization purposes only. A *p*-value < 0.05 was considered statistically significant. Data are presented as mean ± standard deviation (SD).

## Data Availability

The original contributions presented in this study are included in the article/Supplementary Material. Further inquiries can be directed to the corresponding authors.

## Supplementary Materials

The following supporting information can be downloaded at https://www.mdpi.com/article/10.3390/ijms262210832/s1.
