# Supplementary material for: Modulation of AMPK/NLRP3 Signaling Mitigates Radiation-Induced Lung Inflammation by a Synthetic Lipoxin A4 Analogue
Source: Int J Mol Sci. 2025 Nov 7;26(22):10832. doi: 10.3390/ijms262210832 (PMC12652067; doi:10.3390/ijms262210832)
Supplement: Supplementary file 1 [file ijms-26-10832-s001.zip › ijms-3772824-supplementary.pdf]

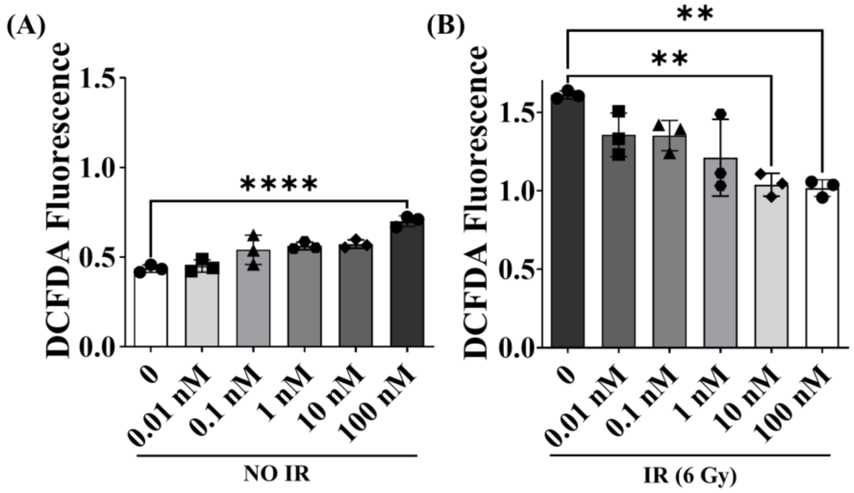

Figure S1. CYNC-2 attenuates radiation-induced intracellular reactive oxygen species (ROS) accumulation as assessed by a DCFDA-based oxidative-stress assay in L132 cells. L132 cells were pretreated with CYNC-2 (10 nM) for 2 hours and then exposed to 6 Gy of ionizing radiation. Intracellular ROS levels were quantified using DCFDA fluorescence intensity measurements to evaluate oxidative stress responses. (A) Non-irradiated control group; (B) Irradiated group; CYNC-2 treatment significantly reduced ROS levels in irradiated cells compared with those without CYNC-2 treatment, indicating its antioxidant and radioprotective effects. Data are presented as mean ± standard deviation (**p < 0.01, ****p < 0.0001 vs. IR alone).

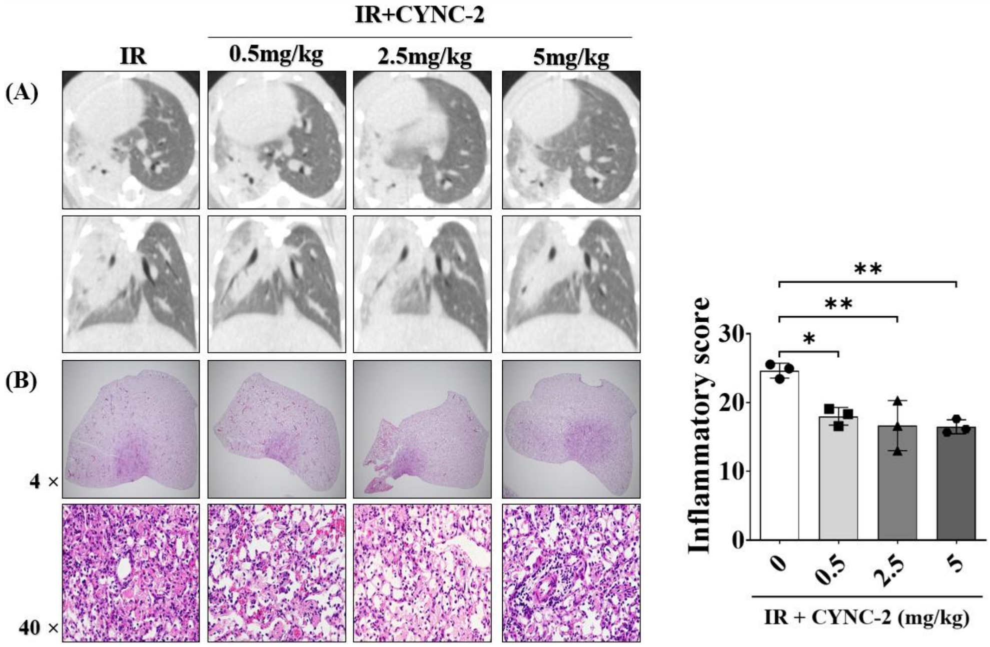

Figure S2. Dose–response evaluation of CYNC-2 in a murine model of radiation-induced lung inflammation. Mice received 0.5, 2.5, or 5 mg/kg CYNC-2 for two weeks post-irradiation. (A) Representative micro-computed tomography (micro-CT) images (top: horizontal view; bottom: trans-axial view). (B) Lung sections

stained with hematoxylin and eosin (H&E). Histological analysis of lung tissues revealed significant reductions in inflammation in all CYNC-2–treated groups compared with irradiated controls (*P < 0.05, **P < 0.01), with no dose-dependent differences observed. Data are presented as mean ± standard deviation (n = 3 per group).

Table S1. Stability comparison of native Lipoxin A4 and CYNC-2.

Compound	Time / Temperature	0 hour	3 hour	6 hour	9 hour	1 day	2 day
Native Lipoxin A4	-70 °C	91.379 %	91.849 %	92.545 %	89.192 %	89.112 %	88.377 %
	-20 °C		89.865 %	90.306 %	90.061 %	90.550 %	90.671 %
	4 °C		91.169 %	83.338 %	81.559 %	87.395 %	87.097 %
CYNC-2	-70 °C	95.688 %	95.545 %	95.511 %	95.123 %	95.642 %	95.503 %
	-20 °C		95.629 %	95.351 %	95.307 %	94.922 %	95.491 %
	4 °C		96.172 %	95.832 %	95.365 %	95.132 %	95.174 %

Table S2. The parameter description of the flexiVent™ system measurements.

Abbreviations	Parameter	Description
IC	Inspiratory Capacity	Amount of air that can be inhaled after the end of a normal expiration.
Rrs	Resistance of the respiratory system	Dynamic resistance quantitatively assesses the level of constriction in the lungs.
Crs	Compliance of the respiratory system	Compliance (also known as dynamic compliance) describes the ease with which the respiratory system can be extended. In a subject with intact chest walls, it provides a characterization of the overall elastic properties that the respiratory system needs to overcome during tidal breathing to move air in and out of the lungs.
Ers	Elastance of the respiratory system	Elastance captures the elastic stiffness of the respiratory system at the ventilation frequency. If measured under closed-chest conditions, it includes a contribution from the lung, the chest walls, and the airways. Elastance is the reciprocal of compliance and vice versa.
Rn	Newtonian Resistance	Parameter of the Constant Phase Model which represents the resistance of the central or conducting airways.
G	Tissue Damping	Parameter of the Constant Phase Model closely related to tissue resistance and reflects the energy dissipation in the alveoli.
H	Tissue Elastance	Parameter of the Constant Phase Model closely related to tissue elastance and reflects the energy conservation in the alveoli.
Cst	Quasi-static Compliance	Quasi-static compliance is a classic parameter extracted from a PV curve. If measured under closed-chest conditions, it reflects the intrinsic elastic properties of the respiratory system (i.e. lung+chest wall) at rest.

A	Tissue damping-related parameter	Reflects tissue resistance or damping (energy dissipation within lung parenchyma)
Area	Hysteresis area (of P-V loop)	Area between inflation and deflation limbs of the pressure–volume curve; indicator of lung elasticity and surfactant function

Table S3. The clinicopathologic characteristics of lung cancer patients analyzed in this study.

Patient No.	Pathology	Radiotherapy Dose	Radiotherapy Period	Radiotherapy to Surgery interval	Concurrent chemotherapy (before RT/after RT)	Initial stage	Postop Stage
# 1	Adenocarcinoma	50 Gy in 25 fractions	38 days	61 days	Taxotere /Cisplatin (6cycles/4cycles)	icT2N2M0	ypT2N0M0
# 2	Adenocarcinoma	54 Gy in 30 fractions	49 days	51 days	Taxotere/Cisplatin (4cycles/0cycles)	icT2N2M0	ypT2N2M0
# 3	Squamous cell carcinoma	50.4 Gy in 28 fractions	41 days	42 days	Taxotere/Cisplatin (7cycles/0cycles)	icT4N1M0	ypT1N0M0
# 4	Small cell lung cancer	54 Gy in 27 fractions	37 days	48 days	Etoposide/Cisplatin (4cycles/0cycles)	icT2N2M0	ypT0N0M0

Table S4. Primer sequences used for real-time polymerase chain reaction.

Gene	Forward (5′ →3′)	Reverse (5′ →3′)
IL-18	CAGCCTAGAGGTATGGCTGT	TCATGTCCTGGGACACTTCTC
IL-10	TGAGAACAGCTGCACCCACTT	ATCTCCGAGATGCCTTCAGC
NLRP3	CACCTGTTGTGCAATCTGAAG	GCAAGATCCTGACAACATGC
GAPDH	TGATGACATCAAGAAGGTGGT	TCCTTGGAGGCCATGTAGGCC

Cycling conditions: Denature: 95°C for 30 s, 95°C for 4 min, followed by 35 cycles of 95°C for 10 s, 55–61°C for 15 s and 72°C for 15 s
